# Methylene blue protects hair follicle stem cells from oxidative and metabolic stress to enhance hair regeneration

**DOI:** 10.18632/aging.206376

**Published:** 2026-05-05

**Authors:** Kavitha Sadashivaiah, Huijing Xue, Kan Cao

**Affiliations:** 1 Department of Cell Biology and Molecular Genetics, University of Maryland, College Park, MD 20742, USA

**Keywords:** methylene blue, minoxidil, HFSCs, Wnt/β-catenin, GLP-1 RA

## Abstract

Hair follicle stem cells (HFSCs) are vital for maintaining hair growth and regeneration. Their dysfunction, often caused by oxidative stress or metabolic imbalance, leads to hair loss and scalp aging. β-catenin is a key protein in the Wnt signaling pathway, and its activity is crucial for HFSC maintenance and regeneration. In this study, we show that methylene blue (MB), a mitochondrial-targeted antioxidant, promotes HFSC proliferation and viability by both reducing reactive oxygen species (ROS) and activating the β-catenin signaling pathway. Functional tests demonstrated that MB significantly sped up wound healing in HFSC cultures, indicating enhanced regenerative ability. Although co-treatment with antioxidants like vitamins A and C improved ROS scavenging, it surprisingly reduced MB-induced β-catenin activation. Conversely, minoxidil, a clinically approved hair growth stimulant, synergized with MB to further activate β-catenin signaling and enhance HFSC viability. Lastly, exposure to glucagon-like peptide-1 receptor agonists (GLP-1 RAs) resulted in a dose-dependent decrease in HFSC survival, consistent with emerging reports of treatment-related hair loss. Remarkably, pre-treatment with MB protected HFSCs from GLP-1 RA–induced metabolic stress and premature cell death. Overall, these findings identify methylene blue as a multifunctional therapeutic candidate that reduces oxidative and metabolic stress while supporting HFSC–mediated hair regeneration.

## INTRODUCTION

Methylene blue (MB) is a century-old drug known for its potent antioxidant properties [1, 2]. MB easily permeates biological membranes within cellular compartments because it is soluble in both water and organic solvents [3, 4]. It has a long history of medical application and has been used to treat various human diseases, including methemoglobinemia, malaria, vasoplegia, septic shock, cancer chemotherapy–induced toxicity, and neurodegenerative disorders [5–7]. The beneficial effects of MB are partly linked to increased expression of genes regulated by the NF-E2-related factor 2 (Nrf2) and the antioxidant response element (ARE) [1]. Previous studies suggest that MB may extend cellular lifespan, enhance cell proliferation, and decrease p16 expression, a biomarker of aging [3, 5, 8]. Additionally, MB absorbs UVA/B radiation and protects against UVA/B-induced DNA damage in primary human keratinocytes [9]. Furthermore, Xiong et al. demonstrated that MB has cell-proliferative and anti-senescence properties in skin fibroblasts from patients with Hutchinson-Gilford progeria syndrome, highlighting its potential to restore mitochondrial and nuclear function in premature aging cells [10].

The scalp is a distinct anatomical region bordered by the face and neck, characterized by a high density of terminal hair follicles and abundant sebaceous glands [11]. The formation of hair follicles during development depends on a dynamic exchange of molecular signals between epithelial and mesenchymal cells, establishing a lifelong cycle of hair growth and renewal [12]. This regenerative process is driven by hair follicle stem cells (HFSCs), a population of multipotent adult stem cells located in the follicular bulge [13, 14]. HFSCs express hallmark stem cell markers, notably CD34 and K15 [15], and their interaction with dermal papilla cells is essential for maintaining stem cell activity and guiding new hair formation [16, 17]. However, oxidative stress, caused by excessive production of reactive oxygen species (ROS) from environmental stressors like UV exposure and bacterial infection, can disrupt these delicate cellular interactions. Over time, this stress contributes to scalp skin aging, follicle degeneration, and premature hair loss [11].

The maintenance of HFSCs and the processes of hair follicle development, cycling, and regeneration depend on intricate cellular signaling networks. Among these, the Wnt/β-catenin pathway serves as a pivotal regulator that drives HFSC activation and initiates hair regeneration [18]. Oxidative stress influences the function of β-catenin. At very low levels of oxidative stress, the interaction of β-catenin with FOXO transcription factors creates a protective cellular response to combat oxidative damage [19]. Conversely, higher levels of ROS can also stimulate cell proliferation, apoptosis, cell senescence, and transformation [20, 21]. Additionally, androgenetic alopecia is also characterized by the depletion or dormancy of HFSCs and involves the dysregulation of key signaling pathways, such as Wnt/β-catenin pathway.

Several small, drug-like molecules have been investigated for their ability to stimulate hair growth and prevent baldness, with Minoxidil being the most recognized and widely used [22]. It enhances hair growth by stimulating follicular cells and reducing hair loss, yet many users experience progressive shedding once treatment is discontinued [23]. Despite its popularity, the precise pharmacological mechanisms of Minoxidil remain poorly understood [24]. Research has shown that Minoxidil activates the Wnt/β-Catenin signaling pathway in human dermal papilla cells, offering a molecular explanation for its ability to extend the anagen (growth) phase of hair follicles [25]. Interestingly, emerging clinical observations have shown that GLP-1 receptor agonists (GLP-1 RAs), although highly effective for weight reduction and metabolic control, may be associated with early-onset hair loss or thinning in some individuals [26]. These findings highlight the complex interplay between metabolic and follicular signaling pathways and underscore the importance of identifying protective or restorative agents that can counteract treatment-induced follicular stress.

Building on previous findings of MB’s benefits for human skin cell proliferation and rejuvenation, we hypothesized that MB could promote HFSC proliferation and maintenance via the β-catenin signaling pathway. Here, we also aim to determine if combining MB could synergize with minoxidil to stimulate β-catenin signaling pathway and protect HFSCs from GLP-1–induced oxidative stress and maintain hair follicle health.

## RESULTS

### Methylene blue treatment does not change the expression of key HFSC stemness markers

To assess the effect of MB on HFSCs, we first checked whether MB treatment changes the expression of key stemness markers CD34 and K15. HSFCs were treated with or without 100 nM MB for 0 to 15 days and then analyzed for CD34 and K15 expression by immunofluorescence. The concentration of 100nM of MB was determined based on previous studies [3, 5, 8, 9]. MB treatment did not alter the expression of either CD34 or K15 compared to vehicle-treated control HSFCs, even after prolonged exposure (Figure 1A). Consistent with these results, Western blot analysis after 15 days confirmed that MB had no significant effect on CD34 and K15 levels (Figure 1B, 1C). Moreover, long-term MB treatment (15 days) showed no detectable effect on cell cycle progression (Figure 1D).

**Figure 1:**
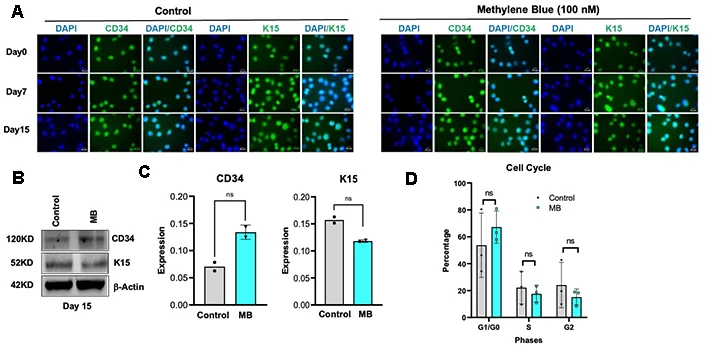
**Methylene blue (MB) treatment does not alter stemness or cell cycle progression in human hair follicle stem cells (HFSCs).** (**A**) Representative immunofluorescence images showing CD34 and K15 expression in control (mock-treated) and MB-treated HFSCs (Scale Bar:10μm). (**B**) Representative Western blot analysis of CD34 and K15 protein levels in control and MB-treated HFSCs. (**C**) Quantification of CD34 and K15 band intensities normalized to β-actin, based on two independent biological replicates. ns: not significant. (**D**) Flow cytometric analysis of cell cycle distribution showing comparable proportions of HFSCs in G0/G1, S, and G2/M phases between control and MB-treated groups (N=3). ns: not significant.

### MB treatment induces proliferation and viability of HFSCs by reducing ROS and increasing β-catenin activation

We next examined the effect of MB on HFSCs proliferation, viability, ROS levels, and β-catenin activation. MB treatment significantly enhanced HFSC proliferation and viability (Figure 2A, 2B). Under physiological conditions, low levels of endogenous ROS transiently activate the HFSC niche and facilitate wound healing; however, excessive ROS accumulation causes oxidative stress, leading to stem cell damage [27]. Flow cytometry analysis after 12–15 days of MB treatment revealed a significant reduction in endogenous ROS compared with vehicle-treated controls (Figure 2C). Since β-catenin is known to regulate HFSC proliferation and differentiation [28], we further assessed the activation status of β-catenin in HFSCs following MB treatment. MB increased β-catenin activation in HFSCs, as shown by elevated nuclear β-catenin levels and enhanced signaling even after 15 days of treatment (Figures 2D, 2E). Collectively, these results suggest that MB promotes HFSC proliferation and survival, potentially by reducing oxidative stress and activating β-catenin-mediated signaling.

**Figure 2:**
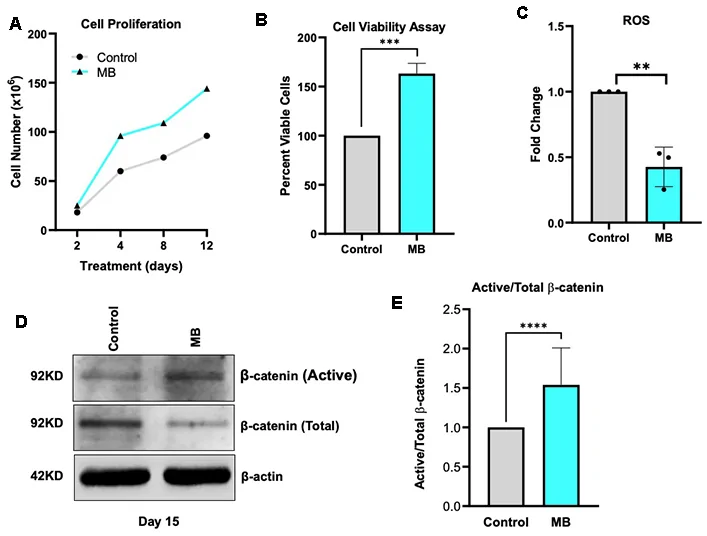
**MB treatment upregulates β-catenin signaling in HFSCs.** (**A**) Growth curve of control and MB-treated HFSCs over 12 days, showing enhanced proliferation upon MB treatment, determined by manual cell counting. (**B**) Cell viability of Control and MB-treated HFSCs at day 15, assessed by XTT assay. (*** p<0.0001). (**C**) Intracellular ROS levels in control and MB-treated HFSCs at day 15, determined by DCFDA staining and quantified using flow cytometry. (** p<0.0001) (**D**) Representative immunoblots of total and active β-catenin expression in control and MB-treated HFSCs at day 15. (**E**) Densitometric quantification of the active/total β-catenin ratio demonstrates a significant increase following MB treatment. (**** p<0.0001).

### MB treatment speeds up wound healing in HFSCs

Activation of the Wnt/β-catenin signaling pathway plays a crucial role in wound healing by promoting cell proliferation, migration, tissue remodeling, and activating stem cells and growth factor expression [29]. Given that MB increased β-catenin activation in HFSCs, we evaluated its effect on cellular wound repair using a scratch assay, a simplified *in vitro* system of wound healing that mimics collective cell migration and gap closure. MB treatment significantly accelerated wound closure compared to PBS-treated controls, achieving near-complete closure within 36–48 hours (Figure 3A, 3B). These findings suggest that MB enhances cell migration and recovery in HFSCs, likely through β-catenin–mediated activation of regenerative pathways.

**Figure 3:**
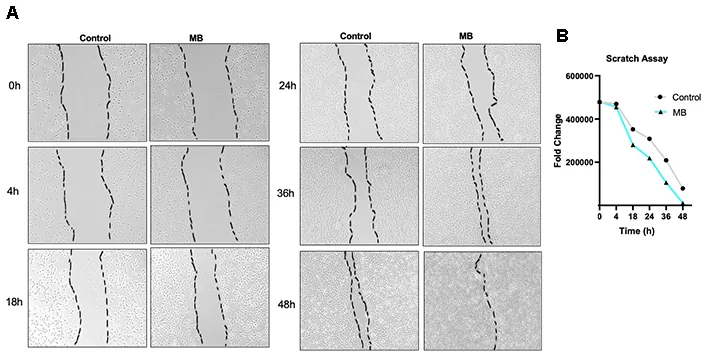
**Methylene blue accelerates wound closure in HFSC monolayer cultures.** (**A**) Representative images of scratch assays in control and MB-treated HFSCs at 0, 4, 18-, 24-, 36-, and 48-hour post-wounding. (**B**) Quantitative analysis of the wound area shows significantly faster closure in MB-treated cells, indicating improved migration and proliferation.

### Co-treatment with vitamins A and C attenuates MB-induced β-catenin activation in HFSCs

Vitamins A and C are well-known antioxidants that protect cells from oxidative damage by reducing ROS-induced oxidative stress [30]. Although their antioxidant role is well established their effects on β-catenin activation remain unclear. Surprisingly, neither vitamin A (100nM) nor vitamin C(100μM), alone or combined with 100 nM MB, increased β-catenin activation beyond what was observed with MB treatment alone after 10 days of treatment (Figure 4A, 4B). These results suggest that co-treatment with antioxidants Vitamins A or C, while likely enhancing antioxidative capacity as previously shown, may paradoxically suppress their β–catenin–mediated activation of HFSC proliferation and function.

**Figure 4:**
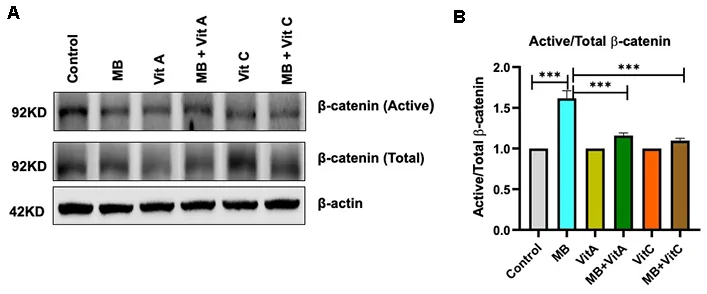
**The individual and combined effects of MB, Vitamin A (Vit A), and Vitamin C (Vit C) on β-catenin expression in HFSCs.** (**A**) Representative immunoblots of total and active β-catenin in HFSCs treated for 15 days in Control, MB (100nM), Vit A (100nM), MB (100nM) +Vit A (100nM), Vit C (100μM), and MB (100nM) +Vit C(100μM) treated HFSCs for 10 days.(**B**) Densitometric quantification of active/total β-catenin ratios from (**A**) shows a significant increase following MB treatment alone. Neither Vit A nor Vit C, alone or in combination with MB, further enhances β-catenin activation beyond MB treatment. Notably, β-catenin activation is partially suppressed in the MB + Vit A and MB + Vit C groups. (***p<0.0001).

### Minoxidil improves hfsc viability and synergistically activates β-Catenin with MB treatment

Minoxidil is a widely used hair growth stimulant that promotes follicular regeneration by activating the Wnt/β-catenin signaling pathway in human dermal papilla cells [25]. To investigate its effects on HFSCs, we treated HFSCs with 1, 2.5, and 5 μM minoxidil for 5 days [24]. Only 1 μM minoxidil increased HFSC viability after 5 days compared to vehicle-treated controls (Figure 5A). Furthermore, combined treatment with 100 nM MB and 1 μM minoxidil synergistically enhanced β-catenin activation in HFSCs, as shown by significantly higher expression of active β-catenin proteins (Figure 5B, 5C). These findings suggest that minoxidil’s hair growth–promoting activity is at least partly mediated through β-catenin activation and that co-treatment with MB could further amplify this regenerative effect.

**Figure 5:**
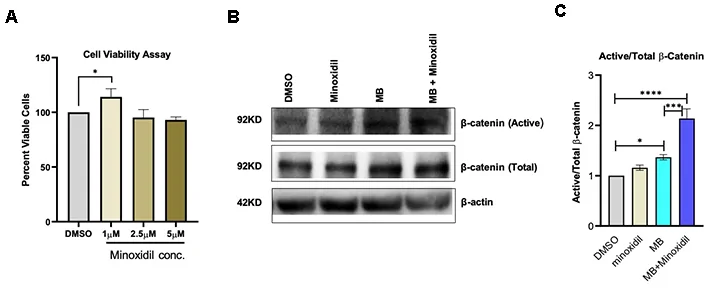
**MB promotes β-catenin activation and exhibits synergistic effects with minoxidil in HFSCs.** (**A**) HFSCs were treated with different concentrations of minoxidil (1 μM, 2.5 μM, and 5 μM) for 5 days, and cell viability was assessed using the XTT assay. The 1 μM concentration of minoxidil showed the most significant benefit for HFSC growth and was selected for subsequent experiments. (**B**) Representative immunoblots showing total and active β-catenin levels in HFSCs treated for 15 days with 1 μM minoxidil, with or without MB co-treatment. (**C**) Densitometric quantification of active/total β-catenin ratios from (**B**) reveals significant activation by MB alone and further synergistic enhancement in the MB + minoxidil combination group. (*p<0.0101, *** p<0.0001, and **** p<0.0001).

### Methylene blue protects HFSCs from GLP-1 RA–induced metabolic stress

Recent clinical observations suggest that, although glucagon-like peptide-1 receptor agonists (GLP-1 RAs) are effective for weight management, they may also cause premature hair loss in some individuals, possibly due to follicular stress or disruption of stem cell signaling [31]. To explore this mechanism *in vitro*, we first examined GLP-1 receptor (GLP-1R) expression across various cell types and confirmed its presence in HFSCs (Figure 6A). This indicates the potential responsiveness of HFSCs to GLP-1 RAs. Treatment of HFSCs with increasing GLP-1 RA concentrations (250–1000 nM) for 5 days [32] caused a dose-dependent reduction in cell viability (Figure 6B, yellow bars), consistent with metabolic stress–induced cytotoxicity. Notably, pre-treatment with 100 nM MB for one week followed by co-treatment with MB markedly preserved HFSC viability under GLP-1 RA exposure, even at 1000 nM (Figure 6B, blue bars, and Figure 6C). These results demonstrate that MB protects HFSCs from GLP-1 RA–induced metabolic stress, likely by maintaining mitochondrial integrity and sustaining β-catenin–mediated regenerative signaling.

**Figure 6:**
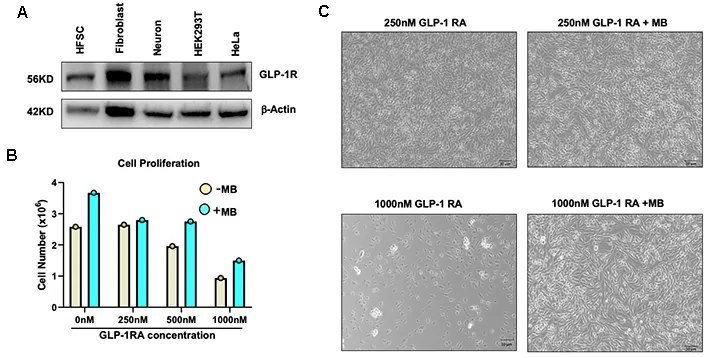
**MB protects HFSCs from glucagon-like peptide-1 receptor agonist (GLP-1 RA) induced cytotoxicity.** (**A**) Representative Western blot showing GLP-1R expression across different cell types. (**B**) HFSCs were cultured on precoated dishes and treated with MB or vehicle for up to 7 days, followed by re-culturing with varying concentrations of GLP-1R agonists for 5 days. Cell proliferation was assessed by manual cell counting. (**C**) Representative bright field images of (**B**).

## DISCUSSION

The scalp and hair follicles are constantly exposed to ultraviolet (UV) radiation, which can cause oxidative stress, DNA damage, and premature aging of follicular stem cells [11]. Traditional sunscreens mainly use inorganic filters like zinc oxide and titanium dioxide or chemical absorbers such as oxybenzone, which reflect or absorb UV light at the stratum corneum but do not provide protection once UV photons pass through the epidermis [9, 33]. As a result, there is increasing interest in identifying bioactive compounds that can neutralize UV-induced oxidative damage within the skin, rather than merely reflecting radiation externally. MB is an agent that was originally synthesized in 1876 and approved by the FDA for medical use [9, 34]. It acts as a mitochondria-targeted antioxidant with well-documented effectiveness in reversing aging features in both progeria and normal human skin cells. Previous research by us and others has shown that MB shields dermal fibroblasts from UVB-induced DNA damage, enhances mitochondrial function, and increases collagen production [2, 10, 35]. These properties, along with MB’s natural capacity to absorb UV light within the 550–700 nm range, make it a new intracellular UV-blocking compound. In cases of thin hair, where the scalp is directly exposed to sunlight, MB provides a biological layer of protection that works in addition to traditional sunscreens by reducing oxidative stress, safeguarding stem cell health, and supporting the scalp’s regenerative capacity. Collectively, these properties establish MB as an excellent active ingredient for promoting scalp health.

Besides protecting the scalp, we further demonstrated MB's ability to reduce ROS-induced cellular damage in HFSCs. We also showed that MB enhances HFSC proliferation and may have the potential to support wound healing processes, likely by activating β-catenin (Figures 2, 3). To evaluate MB’s combined antioxidant effect, we paired it with vitamins A and C, two well-known ROS scavengers. However, only MB uniquely activated the Wnt/β-catenin signaling pathway, which is vital for HFSC proliferation, maintenance, and hair regeneration (Figure 4). In contrast, neither Vitamin A nor Vitamin C has any positive effects on the Wnt/β-catenin signaling pathway under the same conditions. Surprisingly, co-treatment with Vitamins A or C diminished MB-induced β-Catenin Activation in HFSCs (Figure 4). Indeed, Vitamin A derivatives (retinoids) are known to suppress the β-catenin pathway by promoting its phosphorylation and degradation through interaction with the retinoic acid receptor [36]. At the same time, Vitamin C has complex, context-dependent effects, either inhibiting or activating the Wnt/β-catenin pathway depending on cell type and condition. Meanwhile, MB induced sustained β-catenin activation, along with increased HFSC proliferation and wound-healing capacity. This highlights the unique, dual functions of MB; it not only reduces oxidative stress like traditional antioxidants but also stimulates pro-regenerative β-catenin signaling, a property that retinoids (Vitamin A) and Vitamin C either lack or oppose.

Beyond its intrinsic bioactivity, MB also enhances the performance of existing hair-growth stimulants. Minoxidil, an antihypertensive drug later reformulated for topical use after its unexpected association with hypertrichosis, has long been used to treat androgenetic alopecia and promote hair regrowth [22, 37, 38]. Recent studies show that minoxidil activates the Wnt/β-catenin pathway in dermal papilla cells, consistent with our observation that minoxidil increases β-catenin activation in HFSCs (Figure 5). Significantly, co-treatment with MB significantly amplified this effect, indicating a synergistic interaction that enhances stem-cell proliferation and signaling at lower drug doses, potentially reducing common side effects of Minoxidil, such as scalp irritation, dandruff, or unwanted body hair growth [23, 39], with lower drug exposure.

In addition to environmental stress, metabolic factors and therapeutics such as GLP-1 RAs can impair follicular integrity, leading to premature hair loss. Our study further reveals that MB may protect HFSCs from GLP-1 RA-induced metabolic stress (Figure 6), suggesting a potential mechanism that could contribute to mitigating hair loss, which will require validation in *in vivo* models.

The concentrations used in this study fall within established ranges for *in vitro* experimentation and are intended to model cellular responses rather than reflect direct clinical dosing [32]. It is important to note that *in vitro* dosing does not directly translate to physiological exposure levels *in vivo*. Therefore, future studies will be necessary to determine pharmacokinetics, optimize dosing strategies, and define therapeutic windows in relevant *in vivo* models before clinical application can be considered.

A healthy scalp provides the essential “soil” for hair growth, while HFSCs act as the regenerative “seeds” that enable follicle renewal and regrowth. By protecting the scalp microenvironment and supporting HFSC function, MB reinforces both crucial aspects of a healthy hair ecosystem. Additionally, MB’s broad antimicrobial properties may help restore a balanced scalp microbiome, which is increasingly viewed as a factor in scalp conditions like dandruff, seborrheic dermatitis, and atopic dermatitis [40–42]. These findings position MB as a *versatile* molecule that defends the scalp and hair follicles, complements conventional treatments such as minoxidil, and shields HFSCs from metabolic and pharmacologic challenges, fostering a resilient, healthy scalp environment.

## MATERIALS AND METHODS

### Cell culture and antioxidant treatment

The HFSC human frontal scalp region, extracted from the hair follicle bulge, was purchased from Celprogen Inc. (36007-08). HFSCs were maintained in Celprogen’s Human Hair Follicle Stem Cells Complete Growth Medium (M36007-08S) and subcultured every 48 to 72 hours on plates coated with Human Hair Follicle Stem Cells Extracellular Matrix. Methylene blue (MB; #NDC 54288-147-01) was dissolved in PBS and added to the HFSC medium at a final concentration of 100 nM. Vitamin A was applied at 100 nM, and Vitamin C was applied at 100 μM in the treatment.

### Cell proliferation

The desired number of cells (0.1 million) was seeded onto ECM-coated 6-well plates on day 0. They were sub-cultured every 3 days using 0.25% trypsin-EDTA (#25200056) for detachment, and cell counts were recorded for both control and MB treatment (100 nM) over a period of two weeks.

### Immunofluorescence

For immunofluorescence, cells were seeded onto ECM-coated 4-well chamber slides after 24 hours, washed twice with PBS, and fixed with 4% paraformaldehyde (PFA) for 20 minutes at room temperature. They were then permeabilized with 0.5% Triton in PBS for 5 minutes. Cells were washed twice with 1X TBS buffer and blocked with 4% BSA in TBS for one hour at room temperature. Next, cells were incubated with primary antibodies in 4% BSA in TBS overnight at 4° C. The following day, after five washes with TBS, cells were incubated with secondary antibodies diluted in 4% BSA in TBS for one hour. Cells were then washed five times with 1X TBS and mounted with Antifade Mounting Medium with DAPI (Vectashield #H-1200). The primary antibodies used for immunofluorescence included: CD34 (1:500, Santa Cruz-7324 IgG1 κ mouse monoclonal) and K15 (1:500, Santa Cruz-47697 IgG1 κ mouse monoclonal). The secondary antibody used was Alexa Fluor 488 donkey anti-mouse IgG (1:1000, Invitrogen).

### Oxidative stress assay

The Cellular ROS Assay kit (Abcam, ab113851) was used to assess oxidative stress. Cells were dissociated using 0.25% trypsin-EDTA, counted (2×10^^5^ cells for staining), and rinsed with 1× PBS. In 1× BPS buffer, a 12.5 μM DCFDA solution was prepared, and the cells were resuspended in 300 μL of this solution. A buffer-only control sample was included, and the cells were incubated in the dark at 37° C for 30 minutes with intermittent shaking. Flow cytometry was performed using a FACS Canto (BD), and data were analyzed with FlowJo software.

### Western blotting

Whole-cell lysates for immunoblotting were prepared by incubating cells for 10 minutes on ice in 1X Cell Lysis Buffer (Stock concentration 10X, Cell Signaling # 9803S) containing Phosphatase Inhibitor Cocktail 2 (Millipore Sigma # P5726) and PMSF (100 mM stock, 1 mM working concentration) from Cell Signaling. Sample buffer containing 3X Blue Loading Buffer (Cell Signaling # 56036S) and a reducing agent, 1.25M DTT (Cell Signaling #14265S), was added. After incubating on ice for 10 minutes, cells were collected using a cell scraper and centrifuged for 10 minutes at 15000g. Only the supernatant, containing the proteins, was transferred into a fresh tube, and protein concentration was measured using a BCA kit. Then, 20 μg of protein samples were boiled for 5 minutes at 95° C and loaded onto 10% polyacrylamide gels (Bio-Rad), followed by transfer onto 0.45 μm pore-size nitrocellulose membranes (Bio-Rad) using the Turbo blot (Bio-Rad). The membranes were blocked with 5% milk for 1 hour at room temperature. They were incubated overnight at 4° C with primary antibodies, then probed with secondary antibodies for 1 hour at room temperature, followed by ECL development and imaging (Bio-Rad). The primary antibodies used for immunoblotting include: Active β-Catenin (Cell Signaling, 1:1000); Total β-Catenin (Cell Signaling, 1:1000); and β-actin (Sigma-Aldrich, 1:5000).

### Cell cycle analysis

Cells were harvested with 0.25% trypsin and washed once with PBS. Cell pellets were resuspended in 100 μL of 1× PBS per 1–2 × 10^6 cells, followed by vigorous vortexing. Ice-cold 70% ethanol (1 mL) was then added dropwise while gently vortexing, and the samples were incubated at 4° C for 1 hour. After one PBS wash, cells were treated with RNase A (1 μg/μL in 1× PBS) and incubated at 37° C for 30 minutes. Propidium iodide (5 μg; Invitrogen) was then added, and samples were incubated for an additional 30 minutes at 37° C. Flow cytometry was performed on a BD FACSCanto II, and data were analyzed using FlowJo software.

### XTT assay (Cell Viability Assay)

Tetrazolium salt XTT, sodium 3′-[1-[(phenylamino)-carbony]-3,4-tetrazolium]-bis(4-methoxy-6-nitro) benzene-sulfonic acid hydrate, was used for the cell viability assay. Control and pretreated HFSCs were seeded into a 96-well plate at 100 μL per well. After 24 hours, 70 μL of the XTT working reagent was added to each well, and the plates were incubated at 37° C for 4 hours in a CO_2_ incubator. Absorbance was then read at 590 nm.

### Scratch assay

Control and pretreated HFSC cells were seeded in a precoated 12-well plate and incubated until the monolayer reached 75–80% confluence. A scratch was made in the monolayer using a 200 μL pipette tip. The monolayer was gently washed to remove detached cells, then fresh medium was added. Images of the scratch were taken at the start and at regular intervals, and the rate of cell migration was measured.

### Minoxidil treatment

The target number of cells (0.1 million) was seeded onto ECM-coated plates on day 0. The cells were treated with MB or a control for 7 days and sub-cultured at different concentrations of Minoxidil (#J61803.03, Thermo Scientific) with or without MB for up to 5 days. Cell viability was assessed using XTT. Cell lysates were prepared to measure the expression of Active and Total β-Catenin.

### GLP-1 RA treatment

The required number of cells (0.1 million cells) was seeded onto ECM-coated 6-well plates on day 0. The cells were treated with MB or a control for 7 days, then subcultured with various concentrations of GLP-1 RA (#G8048, Sigma-Aldrich) with or without MB for up to 5 days. Cell counts were taken from both treated and untreated cells.
